# Extracellular Acetylated Histone 3.3 Induces Inflammation and Lung Tissue Damage

**DOI:** 10.3390/biom13091334

**Published:** 2023-08-31

**Authors:** Mario C. Rico, Oscar Perez-Leal, Mary F. Barbe, Mamta Amin, Dennis J. Colussi, Magda L. Florez, Victor Olusajo, Dennise S. Rios, Carlos A. Barrero

**Affiliations:** 1 Pharmaceutical Sciences Department, Temple University School of Pharmacy, Philadelphia, PA 19140, USA; mario.rico@temple.edu (M.C.R.); operez@temple.edu (O.P.-L.); dennis.colussi@temple.edu (D.J.C.); magda.florez.lozang@temple.edu (M.L.F.); volusajo@outlook.com (V.O.); 2 Center for Translational Medicine, Lewis Katz School of Medicine, Temple University, Philadelphia, PA 19140, USA; mary.barbe@temple.edu (M.F.B.); mamta.amin@temple.edu (M.A.); 3 Angel Laboratory, Cali 760046, Colombia; dsriosr@unal.edu.co

**Keywords:** histones, COPD, HDAC, H3.3, inflammation, hyperacetylations, cytotoxicity, alveolar damage, cytokines

## Abstract

Extracellular histones, part of the protein group known as damage-associated molecular patterns (DAMPs), are released from damaged or dying cells and can instigate cellular toxicity. Within the context of chronic obstructive pulmonary disease (COPD), there is an observed abundance of extracellular histone H3.3, indicating potential pathogenic implications. Notably, histone H3.3 is often found hyperacetylated (AcH3.3) in the lungs of COPD patients. Despite these observations, the specific role of these acetylated histones in inducing pulmonary tissue damage in COPD remains unclear. To investigate AcH3.3’s impact on lung tissue, we administered recombinant histones (rH2A, rH3.3, and rAcH3.3) or vehicle solution to mice via intratracheal instillation. After 48 h, we evaluated the lung toxicity damage and found that the rAcH3.3 treated animals exhibited more severe lung tissue damage compared to those treated with non-acetylated H3.3 and controls. The rAcH3.3 instillation resulted in significant histological changes, including alveolar wall rupture, epithelial cell damage, and immune cell infiltration. Micro-CT analysis confirmed macroscopic structural changes. The rAcH3.3 instillation also increased apoptotic activity (cleavage of caspase 3 and 9) and triggered acute systemic inflammatory marker activation (TNF-α, IL-6, MCP-3, or CXCL-1) in plasma, accompanied by leukocytosis and lymphocytosis. Confocal imaging analysis confirmed lymphocytic and monocytic/macrophage lung infiltration in response to H3.3 and AcH3.3 administration. Taken together, our findings implicate extracellular AcH3.3 in inducing cytotoxicity and acute inflammatory responses, suggesting its potential role in promoting COPD-related lung damage progression.

## 1. Introduction

Chronic obstructive pulmonary disease (COPD) is a leading cause of disability and death worldwide [1,2,3,4,5]. COPD primarily consists of emphysema and chronic bronchitis. Emphysema involves the destruction of the airways and alveoli, while chronic bronchitis is characterized by airway inflammation and narrowing. In recent decades, the healthcare cost associated with COPD has rapidly increased [6,7]. Although disease management can alleviate symptoms and extend life, there is currently no treatment available to halt disease progression. 

Cigarette smoke remains the primary cause of COPD [8,9], while other risk factors include exposure to occupational dust and chemicals, air pollution, genetic factors, and respiratory infections [10]. A key mechanism underlying the pathogenesis of COPD is the persistent inflammation of the airway [10,11], which can be exacerbated by factors such as continuous exposure to cigarette smoke. Additionally, the imbalance between proteases and anti-proteases, as well as oxidants and antioxidants, plays a role in COPD [11,12]. These processes collectively lead to various pathogenic events, which include inflammation, apoptosis, oxidative stress, damage to extracellular matrix structure, and abnormal tissue remodeling [10,11,12]. A common underlying factor in these pathologic processes is the presence of post-translational modifications (PTMs) in crucial lung proteins, impairing their normal function [11]. These proteins encompass acetyl- and methyltransferases, deacetyl- and demethylases, and histones [4,9,12,13,14]. Among the observed PTMs in COPD, acetylation and methylation are the most prevalent.

We demonstrated that histone 3.3 (H3.3) and the acetylated form of H3.3 were significantly elevated in the lungs of subjects with COPD [13]. Additionally, proteomic studies have revealed PTMs, including acetylation in histones H3 and H4 in lung cells [15,16]. Notably, histones and non-histone proteins can undergo PTMs that influence their function in COPD. These modifications include acetylation and deacetylation mediated by histone acetyltransferases (HATs) and histone deacetylases (HDACs), respectively. Dysregulation of these PTMs and associated enzymes, specifically, impairment of the HDAC2, have been implicated in the pathogenesis of COPD [9,14,15,16]. Under normal circumstances, histones primarily reside within the nucleus and are tightly regulated. Although some histones, such as H1, can remain in the cytoplasm and play different functions [17], most chromatin unbound histones are rapidly degraded. Excess histones are predominantly degraded by proteosomes through site-specific modifications such as phosphorylation and ubiquitination [18,19].

In addition to their nuclear functions, emerging studies suggest that histones can be released into extracellular spaces by activated immune, apoptotic, or necrotic cells. In vivo and in vitro results have shown that histones can exhibit significant toxic and pro-inflammatory activity [20,21,22]. Furthermore, we have discovered that extracellular H3.3 is cytotoxic to human primary lung structural cells in vitro, primarily through a mechanism driven by the disruption of calcium homeostasis, endoplasmic reticulum (ER) stress, and mitochondrial membrane permeability that leads to caspase activation and apoptosis [13]. These findings support previous reports that showed calcium signaling mediated apoptosis in cells exposed to nucleosome histones [11,23].

Histones are crucial components of chromatin and are the building blocks of nucleosomes. These proteins formed octamers consisting of two molecules of each core histone (H2A, H2B, H3, and H4) which package DNA and regulate the gene transcription through modifications that occur in its amino acid sequence [24]. Nucleosome composition can be altered through increased histone acetylation due to the reduced HDAC2 activity and impaired proteasome degradation that is commonly associated with COPD [25]. Moreover, this hyperacetylation of histones has been related to inflammatory gene expression [9,14,26,27]. Therefore, further studies are needed to explore the pathologic effects and potential mechanisms of action of aberrant extracellular histones in lung tissue. 

In this study, we aim to analyze the direct lung toxicity of H3.3 and compare it with its acetylated form in mice, in vivo. Our hypothesis is that abnormal extracellular AcH3.3 contributes to lung toxicity in COPD. Understanding its mechanisms of action could provide new insights into the disease’s pathophysiology and potentially lead to the development of novel therapeutic interventions. By elucidating the intricate involvement of histone modifications in the pathogenesis of COPD, this research has the potential to facilitate the development of precise and targeted therapeutic interventions aimed at ameliorating the adverse effects of this incapacitating disorder.

## 2. Materials and Methods

### 2.1. Reagents

rH2A and rH3.3 proteins were obtained from New England Bio Lab, Ipswich, MA, USA (Cat. #s M2502S, M2507S; respectively). The rAcH3.3 from Active Motif, Carlsbad, CA, USA (Cat. #31289) is a recombinant H3 pan-acetyl synthetic modified histone, containing acetylation at K4, K9, K14, K18 and K23 aa. According to the manufacturer, the rAcH3.3 histone is >95% pure by SDS-PAGE, with a molecular weight of 15,483 Daltons. This was confirmed by high-resolution ESI-TOF mass spectrometry. The difference in the molecular weight between rH3.3 and AcH3.3 is 210 Daltons, due to five acetylations (42 Da each). Cleaved caspase (cCas)-3 and cCas-9 antibodies were obtained from Cell Signaling, Danvers, MA, USA. and biotin antibody, streptavidin, and DAB Substrate Kit (Cat#550880) were obtained from BD Bioscience, San Jose, CA, USA.

### 2.2. Mouse Lung Instillation 

The Institutional Animal Care and Use Committee of Temple University approved this animal protocol. C57BL/6 mice of 8-weeks of age obtained from Jackson’s laboratories were anesthetized using intraperitoneal injection of Ketamine/Xylazine (100/40 mg/g of body weight) and positioned in a rodent-tilted work stand, Hallowell EMC. Each animal was suspended from the upper teeth and the tongue was placed aside for accessibility of the airway. A fiber optic, light source-equipped arm was used to visualize the airway. Proteins were administered with a MicroSprayer^®^ model I-1C (PennCentury™), which was placed into the tracheal opening between the vocal cords. Animals were closely monitored and, under anesthesia, euthanized by exsanguination 48 h after protein instillation. 

### 2.3. Tissue Sample Collection and Blood Cell Counts

Under general anesthesia, total blood was collected by cardiac puncture and mixed with 1/10 *v*/*v* of 3.8% sodium citrate as an anticoagulant. Fifty microliters of anticoagulated blood were analyzed for hematological cell counts using a Hemavet^®^ automatic counter, as previously described [28,29]. Plasma was separated by centrifugation for cytokine measurements. The left lung was clamped and dissected for RNA and protein for future studies; the right lung was fixed by intratracheal infusion with 4% paraformaldehyde solution. After fixation, the upper lobe of the right lung was transferred to a phosphotungstic acid hematoxylin solution (PTAH) for visualization of soft tissue in the micro-CT scanner, using a similar methodology previously described by this group [30]. The lower lobe of the right lung was paraffin embedded for histology (H&E), immunofluorescence (CD3, CD80), and immunohistochemistry (IHC) (cCaspase 3 and 9) studies.

### 2.4. Immunohistochemistry of Histones 

IHC studies were performed to evaluate the effectiveness of the intratracheal instillation of the histones into the alveolar and bronchial spaces. To accomplish this, animals were anesthetized, and histones were instilled as described above. After 30 min, the animals were euthanized, blood was obtained by cardiac puncture and tissues were perfused with PBS. Lungs were extracted, fixed, and paraffin-embedded for sectioning. Tissue slides were deparaffinized, rehydrated and epitope retrieved as previously described [29,31]. Samples were blocked with 10% goat serum in PBS and then incubated overnight with a biotinylated-antiH3.3 antibody. Samples were washed and incubated with streptavidin. The signal was detected using the BD Pharmingen DAB substrate kit, following the manufacturer’s recommendations. 

### 2.5. Lung Tissue Damage Studies 

The right lung was perfused and inflated with a fixative solution and the right main bronchus was isolated by ligation using a suture to maintain the normal structure of the lung. The tissue was paraffinized and sectioned. Stained H&E lung, 5 μm sections were used to determine the accumulation of inflammatory cells, small airway wall thickening, mucus accumulation, alveolar cellular infiltration, and wall destruction. Analysis of the cellularity and thickness of the alveolar wall was performed by taking measurements from multiple pictures of different regions of the lung. Areas with large bronchi or large blood vessels were excluded. To evaluate the ratio of the alveolar tissue over the total lung area, several photographs were transferred into a black-and-white image 8-bit type. Using image J software, a threshold was selected where most of the tissue was acquired, and measurement of the selected area was taken. The measurement was divided by the area (Integrated Density Value IDV/area of the lung tissue).

### 2.6. Micro-CT Analysis of Murine Lung Tissue

Paraformaldehyde (4% in PBS) fixed lung tissue was immersed in 2% phosphotungstic acid, 0.02% potassium permanganate, and 0.1% hematoxylin solution (PTAH) for an average of 5 days in order to visualize soft tissue using micro-CT. PTAH-prepared tissues were scanned in a Skyscan 1172, 12 megapixel, high-resolution image pixel resolution size of 5.89 µm, X-ray source spot size of 300 nm, Al 0.5 mm filter, voltage of 59 kV, current of 167 µA, rotation step of 0.40°, frame averaging of 5. The lung tissue structure was 3D reconstructed using Skyscan N-recon (reconstruction), visualized using CTVox volume rendering software, and analyzed using Skyscan CT-An software. Micro-CT scanning was useful to demonstrate and measure the presence of tissue damage and air trapped inside the lungs. Radiopaque portions of the images were considered pulmonary tissue, or tissue volume, while radiolucent sections were considered alveolar space. Indirect measurement of the lung tissue volume/total tissue volume ratio was calculated using ImageJ software, version 1.53t. 

### 2.7. Apoptosis Measurement

Structural lung cells were examined for apoptosis markers by IHC as described above. Measurement of the ratio of apoptotic-positive/total cells in a 100× magnification field was performed, analyzing a minimum of 5 fields for each lung section per animal to have a representative sample for statistical analysis. 

### 2.8. Inflammatory Markers

Levels of inflammatory cells including neutrophils, macrophages, and lymphocytes and their cytokine mediators were measured in blood. Cytokines including TNF-α, IL-1β, IL 6, IL-8, MIP-2, MCP-1, MIP-1a, MIP-1β, MCP-3, CXCL1, PGE2, IL-12, IL-18, RANTES, and IP-10 were measured using Multiplexed Protein Assay (AssayGate, Inc.; Ijamsville, MD, USA).

### 2.9. Immunofluorescence and Confocal Imaging Analysis

Tissue slides were deparaffinized and dehydrated as explained above. Specific lymphocyte and monocyte/macrophage lung infiltration were evaluated by immunofluorescence measuring CD3 (100244 Biotin anti-mouse CD3) and CD80 (104748 Ultra-LEAFTMPurified anti-mouse CD80) surface markers. Fluorescent detection of the primary antibodies was performed using goat anti-Armenian hamster IgG H&L (Abcam AB173004, Alexa Fluor^®^ 647) and tomato red tagged streptavidin protein. DAPI was used for nuclear staining and anti-podoplanin monoclonal antibody (AF488-conjugated) was used for membrane surface. The lung tissues were then imaged using the Operetta^®^ CLSTM High Content Analysis System (PerkinElmer, Waltham, MA, USA). Specific parameters for tissue detection and identification were established. Data analysis was performed using the Harmony 4.8 software from PerkinElmer.

### 2.10. Statistical Analysis

Differences among groups were statistically analyzed using one-way ANOVA followed by uncorrected Fisher’s LSD Test between groups (each comparison stands alone), *p* < 0.05 was significant. Data are reported as mean ± standard error of the mean (S.E.M.) for each group. Statistical analysis was performed using Prism 9 software, version 9.5.1.

## 3. Results

### 3.1. Histone 3.3 Deposits at the Alveolar Epithelial Lining and the Surface of the Bronchial Lumen

**Figure 1:**
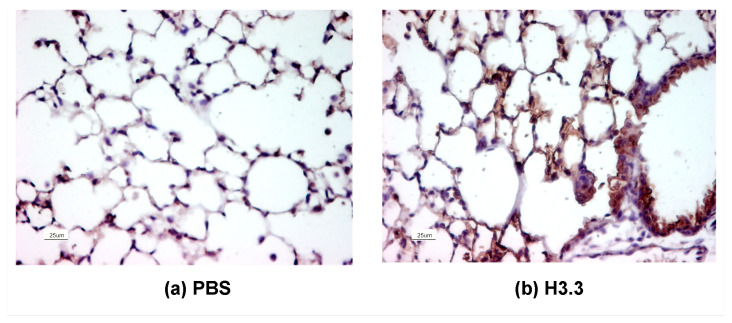
Recombinant histone reaches the alveolar tissue after tracheal instillation. Thirty minutes after tracheal aerosolization of 25 µL of PBS or 25 µL (25 µg) of rH3.3, lungs were flushed with PBS, fixed, embedded, and sectioned for H3.3 immunohistochemistry. Lung instillation of (**a**) PBS or (**b**) H3.3, *n* = 3, 40× magnification, scale bar = 25 μm.

To demonstrate the presence of the proteins at the alveolar tissue after intratracheal instillation, a single dose of 25 µL of recombinant Histone 3.3 (rH3.3) (1 µg/g of body weight in PBS) was administered to mice that were euthanized 30 min after the procedure. A vehicle (25 µL of PBS) was used as a control. Both lungs were paraformaldehyde-fixed, paraffin-embedded, and sectioned. Slides were subjected to immunohistochemistry and tested for H3.3 using a polyclonal antibody. The presence of H3.3 after exposure of the biotinylated antibody at the bronchoalveolar surface confirmed the presence of the rH3.3 post intratracheal administration when compared with control (Figure 1). Animals instilled with PBS showed minimal biotin-antibody-H3.3 staining at the nuclei of the alveolar cells, with no presence in the extracellular space. In contrast, extracellular staining was increased in the mouse lungs instilled with the histones at the alveolar walls and the bronchial lumen. 

### 3.2. Histone 3.3, Particularly When Hyperacetylated, Severely Damage Lung Tissue after 48 h of Direct Instillation

**Figure 2:**
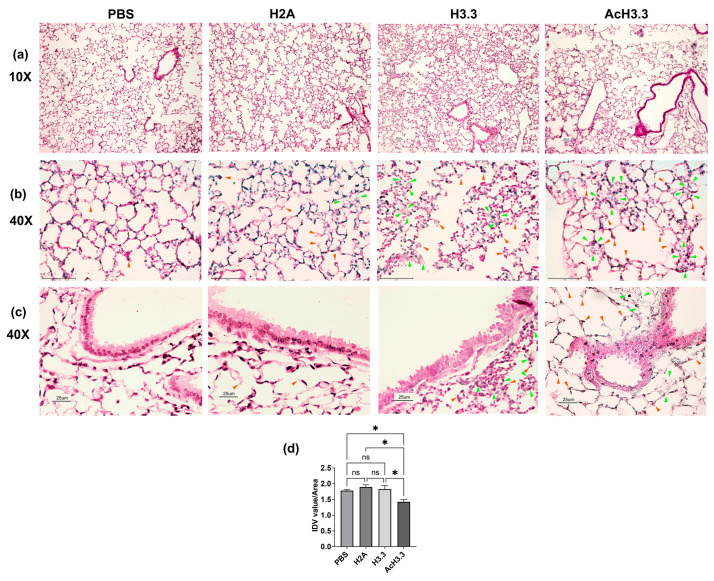
Intratracheal-aerosolized rH3.3 and rAcH3.3 induce structural lung tissue damage. (**a**) Representative photographs of lung tissue stained with hematoxylin and eosin (H&E) at magnifications of 10×, (**b**) parenchyma 40× (Scale bar 75 µm), and (**c**) airway. Lung tissue of H3.3 and AcH3.3 showed cellular infiltration, inflammatory exudates, and alveolar wall rupture. Quantitation of the integrated density value (IDV) area of the lung tissue by ImageJ analysis * *p* < 0.05 AcH3.3 vs. PBS control and vs. H3.3, ns = not significant, *n* = 4–6 per group comparing each group against the other groups (**d**). Green arrowheads point at representative leukocyte infiltration, and orange arrowheads point at alveolar wall damage/rupture (**b**,**c**).

We tested the in vivo toxicity of AcH3.3 in C57BL/6J mice. Thirty-two, 10-weeks old, male mice obtained from Jackson laboratories were randomly separated into four groups, each containing eight animals. Each mouse received an intratracheal dose, in a volume that did not exceed 25 µL of the vehicle (PBS) or 25 µL of the recombinant proteins at 1 µg/g body weight dose of rH2A (the non-cytotoxic histone), rH3.3, or rAcH3.3 (hyperacetylated isoform). After 48 h, the animals were euthanized, and lung tissue was processed for histology and immunohistochemistry analysis. Compared to the control groups (PBS- and rH2A-treated), the thickness of the epithelial airway was increased in the lungs treated with rH3.3 and rAcH3.3. Alveolar wall damage, characterized by enlargement of alveolar space and disruption of structural cells, was also observed. The hematoxylin-eosin staining uncovered a distinct pattern of airway remodeling in both tissues exposed to H3.3 and AcH3.3 when compared to controls. This pattern is characterized by the disruption of alveolar septa in a centrilobular fashion, displaying varying degrees of severity, with the hyperacetylated histone showing more pronounced effects. Furthermore, numerous foci of thickened alveolar septa, along with cells exhibiting enlarged and vacuolated cytoplasm, are evident in the lung samples of H3.3 and AcH3.3. The tissue exposed to H3.3 and AcH3.3 exhibits focal mononuclear inflammatory infiltrates in alveolar septa near the respiratory bronchiole, which are not observed in the controls (Figure 2). Overall, the pathological changes observed were more pronounced in the rAcH3.3 group, compared to the other groups (Figure 2). The lungs instilled with AcH3.3 showed a decrease in the lung tissue/area ratio compared to PBS control (* *p* < 0.05). These indirect measurements were performed using ImageJ software, version 1.53t (Figure 2d). Therefore, both rH3.3 and, more notably, rAcH3.3 induced cytotoxicity and acute lung damage in mice, similar to what is observed in human COPD lungs. 

### 3.3. Micro-CT Scanning and 2D/3D Reconstruction of Murine Lungs Showed a Decrease in Lung Tissue Density

**Figure 3:**
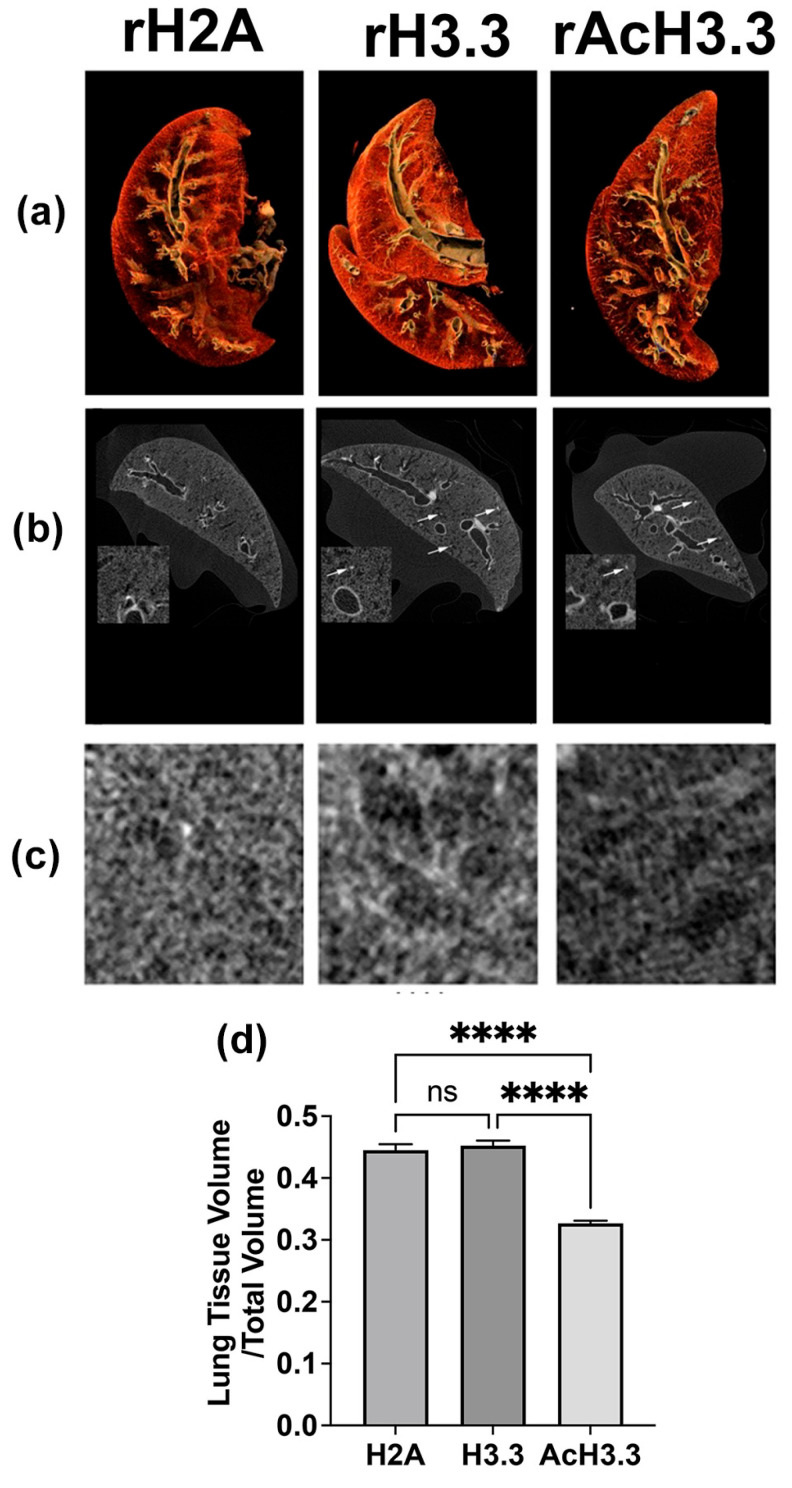
Micro-CT scan reconstruction of the anatomical structure of the rAcH3.3-aerosolized lungs demonstrated alteration of the parenchyma/air space ratio compared to H2A control. (**a**) The 3D reconstruction of the micro-CT images of the lungs aerosolized with histones (CT Vox). (**b**) The 2D sections (i.e., micro-CT slides) of the upper lobe where the main bronchi are located. Inside each panel is a magnification of the lung parenchyma. White arrows are pointing to radiopaque areas observed only in the rH3.3- and rAcH3.3-aerosolized lungs. (**c**) Magnification of the lung parenchyma areas. (**d**) Quantification of the lung tissue/total lung volume. **** *p* < 0.0001 each histone treatment group, ns = not significant. (*n* = 3 per group).

To further assess the extent of lung damage, both 3D and 2D models of the lungs’ architecture were performed by micro-CT scanning. Micro-CT quantification algorithms allowed us to accurately measure the extent of lung destruction and the distribution of air trapping. To visualize soft tissue in the micro-CT, lung tissues were immersed in PTAH (hematoxillin-phosphotungstic solution) for three days, which made soft tissues radiopaque. Multiple sections were acquired (at 5.89 mm^2^ voxel resolution) using the Skyscan^®^1172, ex-vivo Micro-CT instrument. The sagittal section of the 3D reconstruction of the lung micro-CT images is shown in Figure 3a. No significant macroscopic changes were observed in the analyzed lungs. However, upon analysis of small alveolar areas in the 2D micro-CT sections, a decrease in radiopacity was observed in the PTAH-stained tissue, along with sporadic punctual condensations in the lung tissue, in mice treated with rH3.3 and rAcH3.3 (Figure 3b). Furthermore, a significant increase in alveolar air space, accompanied by a reduction in lung tissue volume, was detected in the lungs of mice treated with rAcH3.3 compared to rH2A controls (**** *p* < 0.0001) (Figure 3c,d).

### 3.4. Leucocytosis and Lymphocytosis in Plasma after Instillation of rAcH3.3 

**Figure 4:**
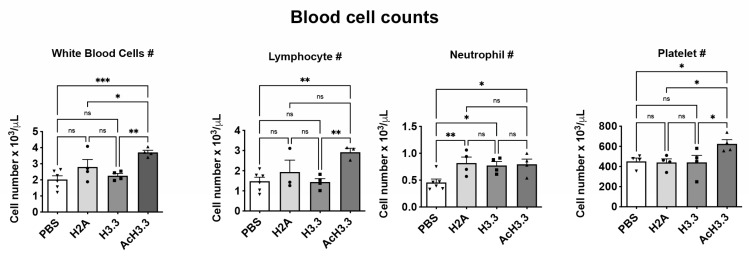
The rAcH.3.3 increases white blood cell counts at the expense of lymphocytes. Automated blood counts from anti-coagulated blood collected by cardiac puncture demonstrated significant leukocytosis and lymphocytosis in mice treated with rAcH3.3 compared to the control (* *p* < 0.05, ** *p* < 0.01, *** *p* < 0.001, ns = not significant). (*n* = 4 per group).

To evaluate systemic changes following tracheal instillation of histones, automated blood counts were measured. The instillation of rAcH3.3 significantly increased the leucocyte count compared with PBS control (** *p* < 0.01). The leukocytosis observed in the rAcH3.3 was primarily at the expense of lymphocytes (* *p* < 0.05). Leukocytes were statistically increased between H3.3 and AcH3.3 (* *p* < 0.01) at the expense of lymphocytes (* *p* < 0.01). No changes in the white blood cell counts were observed with rH3.3 compared with controls. However, an increase in neutrophil count was observed after the tracheal instillation of all histones, including the histone control H2A (Figure 4). A statistically significant increase in platelet count was observed after rAcH3.3 administration (* *p* < 0.05) compared to control and rH3.3. Platelet count was also increased between rAcH3.3 and control (* *p* < 0.05). 

### 3.5. Extracellular rH3.3 and rAcH3.3 Induced Apoptosis at the Damaged Alveolar Tissue

**Figure 5:**
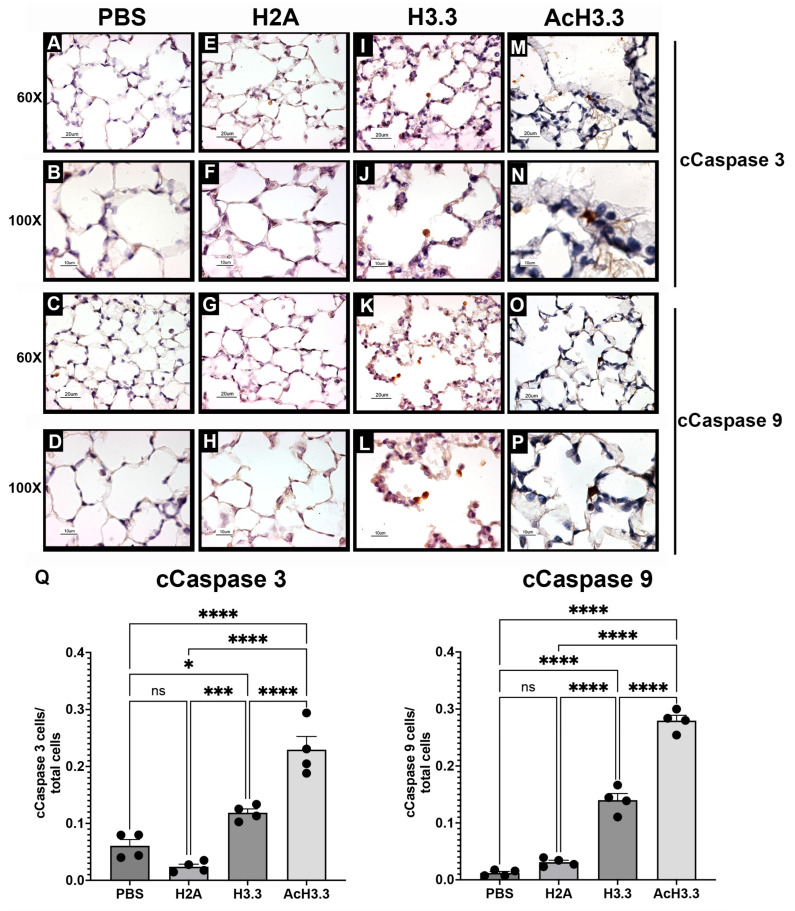
Positive cells of cCaspase-3 and -9 by immunohistochemistry were increased after 48 h of rH3.3- and rAcH3.3-lung aerosolization. (**A**–**D**): PBS, (**E**–**H**): rH2A, (**I**–**L**): rH3.3 and (**M**–**P**): rAcH3.3, (**Q**). Quantitation of positive cells/total cells (* *p* < 0.05, *** *p* < 0.001, **** *p* < 0.0001, ns = not significant). *n* = 4 per group.

There is an increase in apoptotic activity and lung damage at the alveolar wall in COPD. To evaluate the apoptotic activity in the alveolar wall, we investigated the expression caspase cleavage hallmarks of apoptosis, namely cleaved caspase-3 (cCaspase-3) and cleaved caspase-9 (cCaspase-9). The cCaspase-3 is considered the “executioner of apoptosis” while cCaspase-9 activates the cascade of caspases involved in the execution of apoptosis. The tissue exposed to H3.3 and AcH3.3 shows the expression of markers with a distinct cytoplasmic pattern in cells located within the alveolar spaces. The animals treated with rH3.3- and rAcH3.3 exhibited a statistically significant increase in the number of positive pulmonary cells for cleaved caspase-3 and -9, located at the damaged alveolar wall. The cCaspase 3 index was twofold higher with H3.3 and almost fourfold with AcH3.3 compared to PBS. Additionally, the cCaspase 3 level was nearly doubled in AcH3.3 compared to H3.3. Similarly, the cCaspase 9 index was more than tenfold higher with H3.3 and more than twentyfold higher with AcH3.3 compared to PBS. Furthermore, the cCaspase-9 level was nearly twofold higher with AcH3.3 compared to H3.3 (Figure 5). 

### 3.6. High Plasma Levels of Pro-Inflammatory Cytokines Were Found after Instillation of rH3.3 and Even Higher with rAcH3.3

**Figure 6:**
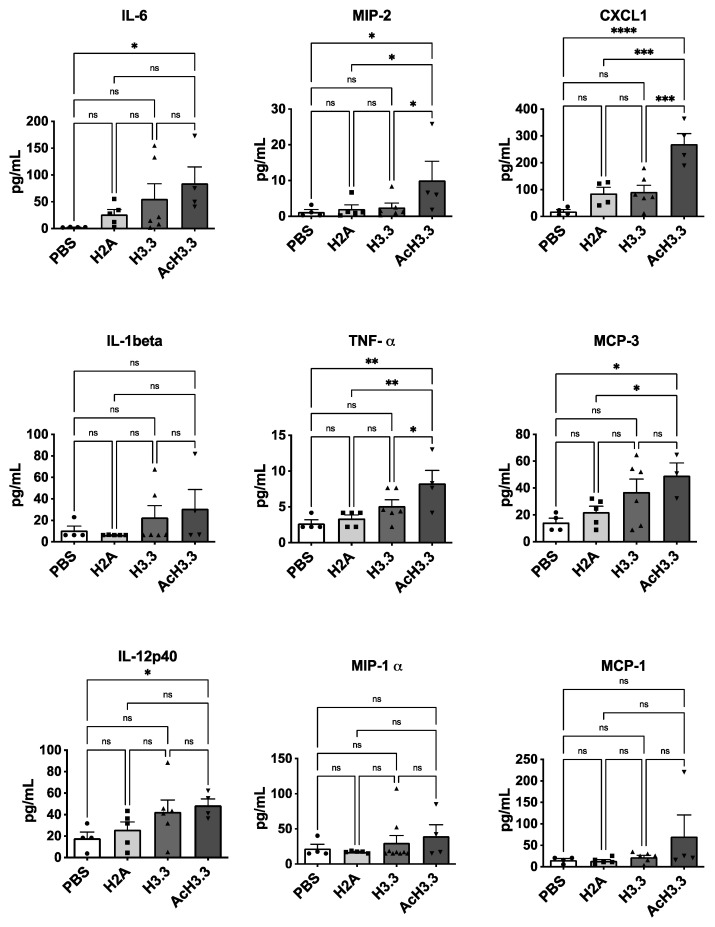
Pro-inflammatory cytokines were increased after rAcH3.3 treatment. Graphic representation of the plasma levels of IL-6, MIP-2, CXCL1, IL-1β, TNF-α, MCP-3, IL-12p40, MIP-1α, and MCP-1 cytokines from animals instilled with PBS (white bars), rH2A (light grey bars), rH3.3 (dark grey bars), and rAcH3.3 (black bars). * *p* < 0.05, ** *p* < 0.01, *** *p* < 0.001, **** *p* < 0.0001, ns = not significant (*n* = 4–6 per group).

Plasma levels of cytokines were measured to assess the systemic effects of lung extracellular histone administration. Following the instillation of rH3.3 and particularly rAcH3.3, several cytokines exhibited increased levels. Proinflammatory cytokines interleukin-6 (IL-6), tumor necrosis factor (TNFα), macrophage inflammatory protein 2 (MIP-2), and interleukin-12 p40 (IL-12p40) were significantly elevated in the plasma of animals treated with rAcH3.3 (** *p* < 0.01, * *p* < 0.05 vs. control). Although plasma levels of interleukin-1 beta (IL-1β) did not reach statistically significant differences, there was a noticeable trend of increased levels following the administration of rH3.3 and rAcH3.3. Additionally, chemokines including chemokine (C-X-C motif) ligand 1 (CXCL1) and monocyte chemotactic protein-3 (MCP3) were increased with histone administration, with a statistically significant increase observed in the AcH3.3 group (* *p* < 0.05). Other cytokines, such as macrophage inflammatory protein-1 alpha (MIP-1α) and monocyte chemotactic protein-1 (MCP-1), showed increased levels after AcH3.3 instillation, but the differences did not reach statistical significance (Figure 6). Cytokines including human interferon-inducible protein 10 (IP-10), interleukin-18 (IL-18), macrophage inflammatory protein-1 beta (MIP-1β), Regulated upon Activation Normal T cell Expressed and Secreted (RANTES), and prostaglandin E2 (PGE2) remained unchanged among the groups. Notably, plasma levels of MIP-2, CXCL1, and TNF-α exhibited a statistically significant increase in the AcH3.3 group compared to H3.3 (* *p* < 0.05).

### 3.7. CD3 and CD80 Protein Levels Were Found Increased after Instillation of rAcH3.3

**Figure 7:**
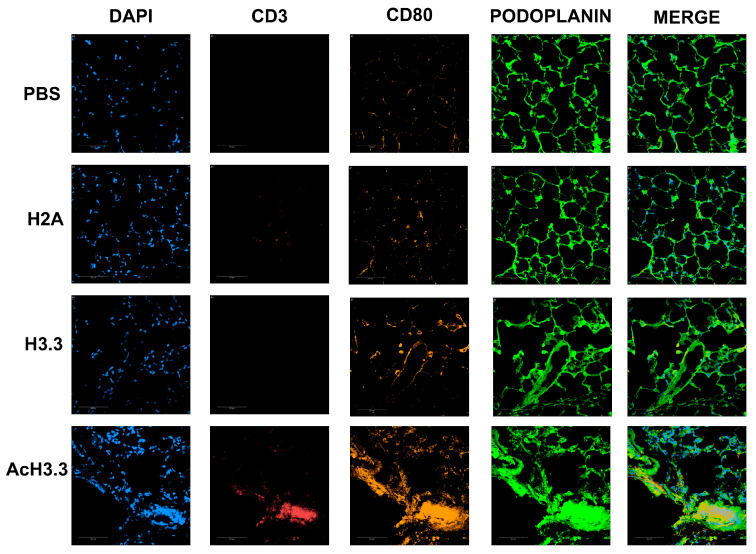
CD3 and CD80 were increased after rAcH3.3 treatment. CD3 (red) and CD80 (orange) protein levels were assessed by immunofluorescence. The membrane surface and nuclei were stained using podoplanin and DAPI, respectively. Scale bar 50 µm.

Specific lymphocyte (CD3) and monocyte/macrophage (CD80) proteins were increased after instillation of AcH3.3, assessed by immunofluorescence. Sections of the lung tissue from the four animal groups were incubated with anti-CD3 and anti-CD80 antibodies to evaluate the presence and localization of the proteins in the lung tissue. Representative pictures from each group are presented in Figure 7. CD3 and CD80 were increased in the AcH3.3 when compared with controls. 

## 4. Discussion

Histone PTMs are critical in chromatin remodeling, gene transcription, and histone degradation. Notably, cigarette smoke has been identified as a significant contributor to the induction of PTMs, thereby promoting the transcription of inflammatory factors in the context of COPD [15,16,32]. Furthermore, alterations in the structure of histones by PTMs can compromise their function [12,33]. Histones with excessive PTMs can impede their degradation and accumulate in the cytoplasm [13,34,35]. Therefore, the accumulated histones are released during apoptosis or cell death and reach the extracellular space, leading to tissue damage. 

In this study, we investigated if the presence of extracellular H3.3 and PTMs of H3.3, particularly hyperacetylation of H3.3, are responsible for acute lung damage. It is known that the concentration of any protein in the pulmonary epithelial lining fluid is 100 times higher than that in the bronchoalveolar lavage fluid (BALF) [36,37]. Previously, we reported that the concentration of H3.3 in the BALF of COPD patients ranges from 50 to over 1000 ng/mL [13]. Therefore, we estimated that the concentration of H3.3 in the epithelial lining fluid ranges from 5 to 100 µg/mL. These concentrations in COPD lungs were taken into consideration when selecting the concentration of H3.3 (1 mg/kg of body weight) used in this study. Using this dosage, we observed a similar distribution of H3.3 in the epithelial lining of the airway (Figure 1) to what we observed in the lungs of COPD subjects [13]. 

The results of this study demonstrated that the acetylated form of H3.3 induces greater structural lung damage compared to unmodified H3.3. Histological studies revealed increased leukocyte infiltration and foci of alveolar thickening in lungs treated with AcH3.3. Micro-CT studies demonstrated a reduction in lung tissue volume. In addition, there were many radiopaque areas in the 2D radiographs from the rH3.3- and rAcH3.3-aerosolized lungs. These areas can be associated with inflammatory cell infiltration and debris from dead cells. These results could be the physiological outcome of peripheral lung tissue destruction. While all evaluated histones significantly increased neutrophil blood count, AcH3.3 also elevated total leukocytes, lymphocytes, and platelets, suggesting an increased acute systemic response. 

Our current study shows that AcH3.3 exhibits significantly greater toxicity than H3.3 in structural lung cells, along with increased cell death concentrations within the alveolar space. These results align with our initial in vitro studies using primary lung cells and hyperacetylated histones extracted from the lungs of COPD patients [13]. Histones are known as DAMP molecules when released into the extracellular space [20]. Although in vivo toxicity has been previously tested using whole nucleosome histones complex, few studies have been performed to evaluate the effects of PTMs in inducing inflammation [38]. Toll-like receptors, such as TLR-2 and TLR-4, have been linked to histone toxic effects and proinflammatory signals [20,38]. However, a change in the molecular charge due to multiple acetylation sites of the N-term of the H3.3 can lead to the activation of additional DAMPs receptors that promote higher apoptosis signals and strongly activate the innate immune response. In addition, inflammasome activation and pyroptosis that can be activated by danger signals could contribute to the increased inflammatory response observed in the AcH3.3-administered mice [39,40]. This phenomenon can be seen by the systemic proinflammatory response induced by AcH3.3 compared to the unmodified H3.3.

The acute systemic cellular response triggered by AcH3.3 was characterized by a pro-inflammatory and chemotactic systemic cytokine profile. Notably, elevated levels of the proinflammatory mediators IL-6, TNF-alpha, and the chemokines CXCL1 and MCP3 were detected in the plasma of the AcH3.3-treated mouse. We confirmed that this systemic activation results in an acute recruitment of immune cells including lymphocytes, monocytes, and macrophages in response to AcH3.3. While previous studies have reported lung structural damage associated with nucleosome proteins [20,41], our findings highlight the specific detrimental effect of a hyperacetylated modified histone, implicating it as a causative factor in lung damage. 

Moreover, the change in the alveolar architecture induced by AcH3.3 was associated with cell death, mediated by active caspase activation, when evaluated at the molecular level. Cleaved caspases 3 and 9 were detected in the disrupted alveolar cells treated with H3.3 and AcH3.3. These findings suggest that alveolar epithelial cells could be more sensitive to the toxicity mediated by extracellular histones, especially H3.3 and its acetylated isoform. These findings are in agreement with our previous in vitro work [13] and by others [35,42] that extracellular histones, specifically H3.3, induce a repetitive calcium flux that alters the mitochondrial membrane potential and promotes the initiation of the caspase cascade [43].

Our research has conclusively demonstrated that both rH3.3 and rAcH3.3 induce cytotoxicity, inflammation, and acute lung damage in C57BL/6J mice. The detrimental effects were more pronounced with rAcH3.3 and are consistent with observations made in human COPD lungs. These findings are of great significance for the advancement of pharmacological therapies aimed at treating COPD. Strategies involving the activation of HDACs or the inhibition of histone acetyltransferases (HATs) can potentially reduce the accumulation of acetylated H3.3. Furthermore, monoclonal antibodies targeting AcH3.3 could mitigate the lung damage caused by extracellular acetylated H3.3. In summary, this study provides valuable insights into the role of extracellular histones in COPD and establishes a foundation for further research investigating their potential as therapeutic targets.

## Data Availability

The data presented in this study are available in the body of the article.
